# Cytosolic Phospholipase A2 in Infiltrating Monocyte-Derived Macrophages Does Not Impair Recovery After Spinal Cord Injury in Female Mice

**DOI:** 10.21203/rs.3.rs-5046064/v1

**Published:** 2024-10-16

**Authors:** Ethan P. Glaser, Timothy J. Kopper, William M. Bailey, Reena Kumari, Hassan K. Kashif, Andrew N. Stewart, John C. Gensel

**Affiliations:** University of Kentucky College of Medicine; University of Colorado Anschutz Medical Campus; University of Kentucky College of Medicine; University of Kentucky College of Medicine; University of Kentucky College of Medicine; University of Kentucky College of Medicine; University of Kentucky College of Medicine

**Keywords:** Spinal cord injury, Cytosolic phospholipase A2, macrophage, myelin, inflammation

## Abstract

Spinal cord injury (SCI) leads to permanent motor and sensory loss that is exacerbated by intraspinal inflammation that persists months to years after injury. After SCI, monocyte-derived macrophages (MDMs) infiltrate the lesion to aid in myelin-rich debris clearance. During debris clearance, MDMs adopt a proinflammatory phenotype that exacerbates neurodegeneration and hinders recovery. The underlying cause of the lipid-mediated MDM phenotype shift is unclear. Our previous work suggests that cytosolic phospholipase A2 (cPLA2) plays a role in the proinflammatory potentiating effect of myelin on macrophages *in vitro*. Cytosolic phospholipase A2 (cPLA2) frees arachidonic acid from phospholipids, generating eicosanoids that play an important role in inflammation, immunity, and host defense. cPLA2 is expressed in macrophages along with multiple other cell types after SCI, and cPLA2 inhibition has been reported to both reduce and exacerbate secondary injury pathology recovery. The role of cPLA2 in MDMs after SCI is not fully understood. We hypothesize that cPLA2 activation in MDMs after SCI contributes to secondary injury. Here, we report that cPLA2 plays an important role in the myelin-induced inflammatory macrophage phenotype *in vitro* using macrophages derived from cPLA2 knockout bone marrow. Furthermore, to investigate the role of cPLA2 in MDMs after SCI, we generated female bone marrow chimeras using cPLA2 knock-out donors and assessed locomotor recovery using the Basso Mouse Scale (BMS), CatWalk gait analysis system, and horizontal ladder task over six weeks. We also evaluated tissue sparing and intralesional axon density six weeks after injury. cPLA2 KO chimeras did not display altered locomotor recovery or tissue pathology after SCI compared to WT chimera controls. These data suggest that although cPLA2 plays a critical role in myelin-mediated potentiation of proinflammatory macrophage activation *in vitro*, it may not contribute to secondary injury pathology *in vivo* after SCI.

## Introduction

Spinal cord injury (SCI) is a devastating condition that results in significant functional deficits and dramatically decreases the quality of life of injured individuals. The initial mechanical trauma to the spinal cord tissue, known as primary injury, is followed by biological events that exacerbate tissue damage. This secondary injury involves a complex biological cascade that includes but is not limited to inflammation, activation of resident neuroglia, peripheral leukocyte infiltration, and oxidative stress. Secondary injury exacerbates tissue damage and hinders recovery thereby limiting endogenous repair [1].

In the injured spinal cord, activated macrophages, made up of resident microglia and infiltrating monocyte-derived macrophages (MDMs), play a large role in the secondary injury response and contribute to both injury and repair [2, 3]. Whether macrophages adopt a reparative, anti-inflammatory or neurodegenerative, pro-inflammatory phenotype is largely due to environmental stimuli. One unique stimulus found in the injured spinal cord is myelin debris. As MDMs arrive in the injured spinal cord, they become the primary phagocytes responsible for myelin debris clearance [4, 5]. Through engulfment of myelin debris, these MDMs begin to phenotypically and morphologically resemble lipid-laden foam cells that are common in other disease states, such as atherosclerosis [5–7]. MDMs also undergo a concomitant proinflammatory phenotypic shift that impairs regeneration [3]. Although it is well established that macrophages clear myelin debris and adopt a proinflammatory phenotype simultaneously, the underlying biological mechanism linking these two processes is unclear.

We previously reported that activation of cytosolic phospholipase A2 (cPLA2) may link myelin with proinflammatory activation [8]. cPLA2 belongs to a superfamily of enzymes called phospholipases that catalyze the hydrolysis of phospholipids into bioactive lipid metabolites. Phospholipases are split into two subcategories, phospholipase 1 and phospholipase (PLA2). The PLA2 family has been extensively studied because it acts on the Sn-2 position of phospholipids to generate free fatty acids (for a review see [9]). Free fatty acids generated from Sn-2 cleavage of phospholipids are largely polyunsaturated and often serve as precursors to eicosanoids and docosanoids. Eicosanoids and docosanoids are a group of bioactive lipid metabolites that have extremely diverse functions and play important roles in the initiation and resolution of the inflammatory response [10].

cPLA2 has been the focus of decades of research because it specifically targets phospholipids containing an arachidonic acid acyl moiety. Arachidonic acid is a polyunsaturated fatty acid that is the precursor for the production of eicosanoids such as prostaglandins, prostacyclins, thromboxanes, and leukotrienes. Eicosanoids have widespread and diverse physiological actions that include vascular permeability, immune cell recruitment, fever, pain, and platelet aggregation [11]. Additionally, cPLA2 is activated by intracellular calcium influx and phosphorylation by several protein kinases, including mitogen-activated protein kinases, Ca2+/calmodulin-dependent protein kinase II, and mitogen-activated protein interacting kinases [12, 13]. Both intracellular calcium and protein kinase activity are triggered by common pathogens and damage-associated molecular patterns found after SCI [14]. cPLA2 activation triggers translocation to nuclear, mitochondrial, lysosomal, and plasma membranes, where it acts on phospholipids to generate free arachidonic acid that is quickly converted to bioactive eicosanoids [15, 16].

From this information, it is not surprising that cPLA2 inhibition is beneficial in numerous disease processes [15, 17–22]. Previous work investigating the role of cPLA2 in SCI using both pharmacologic and genetic tools to inhibit cPLA2 has reported variable effects. cPLA2 was first described in uninjured and injured spinal cord tissue by Ong et al. and Liu et al., respectively [23, 24]. Since then, several reports have assessed the effect of cPLA2 inhibition on SCI outcomes with conflicting results. Most of these reports suggest that cPLA2 inhibition is beneficial to SCI recovery and shows improved locomotor recovery, increased tissue sparing, reduced neuronal death, and enhanced autophagic flux [17, 19, 24, 25]. However, other reports indicate a detrimental effect of cPLA2 inhibition [26]. Previous work has used global-knockout mice and trifluoromethyl ketone analogs (arachidonyl trifluoromethyl ketone (AACOCF_3_) and palmitoyl trifluoromethyl ketone (PACOCF_3_)) to systemically limit cPLA2 activity [18, 27]. We previously documented that cPLA2 is present in macrophages after SCI [8, 28]. We also demonstrated that cPLA2 may play a critical role in myelin-mediated potentiation of proinflammatory macrophage activation *in vitro* using the cPLA2 inhibitor PACOCF_3_. In this report, we first confirmed our previous observations regarding the role of cPLA2 in myelin-mediated potentiation of proinflammatory macrophage activation *in vitro* using cPLA2 KO bone marrow-derived macrophages (BMDMs). Additionally, we generated chimeric mice using cPLA2 knock-out (cPLA2 KO) or wild-type (WT) littermate control donors to interrogate the role of cPLA2 in MDMs after SCI. Although we achieved robust chimerism with dramatically decreased cPLA2 expression in leukocytes and in injured spinal cord tissue, we found no effect of cPLA2 chimerism on locomotor recovery or tissue sparing after SCI.

## Materials and Methods

### Animals:

In these experiments, 2- to 4-month-old female cPLA2 ^−/−^ (cPLA2 KO), cPLA2 +/+ (WT) littermate controls, and Actin-GFP C57BL/6 mice were used as hematopoietic stem cell transplant (HSCT) donors. Three-month-old C57BL/6 mice (Jackson Labs, Bar Harbor, Maine) were used as HSCT recipients. cPLA2 KO mice were previously generated [18], and breeding pairs were generously donated by Dr. Xiao-Ming Xu at the Indiana University School of Medicine and endorsed by Dr. Joseph Bonventre (Harvard University). To reduce the risk of bone marrow rejection or related pathologies (graft versus host disease), cPLA2 KO mice were backcrossed to C57BL/6J females acquired from Jackson Labs. Actin-GFP mice were originally purchased from Jackson Labs but were generously donated by Dr. Ahmed Abdel-Latif at the University of Kentucky. All animals were housed in IVC cages with ad libitum access to food and water. All procedures were performed in accordance with the guidelines and protocols of the Office of Research Integrity and with approval of the Institutional Animal Care and Use Committee at the University of Kentucky. All procedures complied with ARRIVE (Animal Research: Reporting of *In Vivo* Experiments [29]) guidelines.

### Cell Culture:

Bone marrow-derived macrophages (BMDMs) were extracted from the femur and tibia of cPLA2 KO and WT littermate controls as described previously [8]. BMDMs were plated at 0.8–1×10^6^ cells/mL in differentiation media containing Roswell Park Memorial Institute medium (RPMI, Thermo Fisher Scientific, #21870–092) supplemented with 1% pen/strep (P/S, Thermo Fisher Scientific, #5140122), 1% HEPES (Sigma–Aldrich, #83264–100ML-F), 1% GlutaMAX 0.001 (Thermo Fisher Scientific, #35050061), 0.001% β-mercaptoethanol (Thermo Fisher Scientific, #21985023), 10% FBS (Life Technologies, #10082147), and 20% supernatant from sL929 cells (a generous gift from Phillip Popovich, The Ohio State University). Supernatant collected from sL929 cells contains macrophage colony-stimulating factor, which helps to promote bone marrow cell differentiation into macrophages [30]. After 7 days of differentiation, cells were transferred to 12-well plates at a density of 1×10^6^ cells/mL in RPMI containing 1% P/S, 1% GlutaMAX, and 10% FBS. On day 8, cells were stimulated for 24 hours with LPS (50 ng/mL, Invivogen, #tlrl-eblps, standard preparation) and IFN-γ (20 ng/mL, eBioscience #14–8311-63) diluted in N2A growth medium (described below). At the time of stimulation, cells were also treated with myelin debris (50 μL/mL, preparation described below). Twenty-four hours after stimulation, the supernatants were removed and centrifuged at 13,000 RPM (Fischer Scientific AccuSpin Micro R centrifuge), and then this macrophage-conditioned medium (MCM) was either applied directly to immortalized neurons (N2A cells) to measure cytotoxicity or stored at −80°C prior to testing for nitric oxide content using the Griess Reagent KIT (Thermo Fisher Scientific # G-7921) and phenol red-free RPMI.

### Myelin Isolation

Moderate purity myelin (> 95% myelin, with small contributions from the axolemma and other cellular membranes) was prepared as previously described [8]. Brains were collected from C57BL/6 mice and stored at −80°C prior to myelin isolation. Brains were first rinsed and suspended in cold PBS with 1% P/S (PBS/P/S) and homogenized with the loose and tight pestles of a Dounce homogenizer (DWK Life Science, #357544). The resulting suspension was transferred to a 15 mL tube and pelleted at 2000 RPM prior to discarding the supernatant. The pellet was resuspended in PBS/P/S, and then 5 mL of a 30% Percoll solution (Sigma–Aldrich, #P1644–500ML) was gently dispensed below the myelin solution to perform density-graded centrifugation. The layers were then centrifuged at 2000 RPM for 15 minutes at 4°C under gentle acceleration/deceleration. This generated three distinct layers (soluble on top, myelin in middle, and Percoll/cell pellet on bottom). After removing the soluble fraction, the myelin was transferred to a fresh tube, resuspended in 10 mL of distilled water with 1% P/S, and incubated for 10 minutes at 4°C to induce hypoosmotic shock to further separate the membranes. The myelin was then repelleted at 2000 RPM and suspended in PBS/P/S and Percoll to perform a second density gradient centrifugation as described above. The myelin was then suspended and pelleted twice in PBS/P/S to remove residual Percoll and water-soluble contaminants. Isolated myelin was then aliquoted and stored at − 80°C. The final protein concentration of the myelin stock solutions produced by this protocol was 10.23 mg/mL with a standard deviation of 0.282 mg/mL as determined by a BCA Protein Assay Kit (Thermo Fisher Scientific #23225). With the application of myelin debris to BMDMs at 50 μL/mL, cells had a mean dosage of 0.51 mg/mL. Finally, to ensure that our results were not due to endotoxin contamination in our myelin preparations, we tested aliquots from each batch of myelin stimulant (Thermo Fisher Scientific #88282).

### Neurotoxicity Assay

To assess the neurotoxicity of stimulated BMDMs, a mouse neuroblastoma cell line (Neuro-2a or N2A, a gift from Chris Richards, University of Kentucky) was maintained in N2A growth medium consisting of 45% DMEM, 45% OPTI-MEM reduced-serum medium, 10% fetal bovine serum (FBS), and 1% penicillin/streptomycin. N2A cells were plated at a density of 1×10^5^ cells/mL in 96-well tissue culture plates and allowed to proliferate for 48 hours. The neurotoxicity of macrophage-conditioned media (MCM) was evaluated as reported previously [8] using an MTT-based cell growth determination kit according to the manufacturer’s instructions (Sigma–Aldrich CGD1–1KT). Briefly, 24 hours before testing, N2A growth media was replaced with serum-free N2A media to induce differentiation. On the day of testing, this medium was replaced by fresh MCM, and the N2A cells were incubated in MCM for 24 h before thiazolyl blue tetrazolium bromide (MTT (5 mg/ml), 20 μl per well) was added to each well, and the cells were further incubated for 2 h. The tetrazolium ring of MTT can be cleaved by mitochondrial dehydrogenases of viable cells, yielding purple formazan crystals, which were then dissolved in acidified isopropanol solvent. The resulting purple solution was spectro-photometrically measured at 570 nm in an Epoch microplate reader (BioTek Instruments, Inc., Winooski, VT) using 690 nm as the background absorbance. These data are normalized to the nontoxic CTL values to generate a proportional decrease in viability values.

### Reactive Oxygen Species Assay

Macrophage reactive oxygen species (ROS) production was measured using chloromethyl 2′,7′-dichlorodihydrofluorescein diacetate (CM-H2DCFDA) (Invitrogen-gen #C6827) as previously described [8]. In short, BMDMs were cultured and stimulated as described above except in a 96-well plate (1×10^6^ cells/mL). Following the 24-hour stimulation, the supernatants were removed and replaced with a 5 μM solution of CM-H2DCFDA in phenol red-free RPMI with 1% GlutaMAX and penicillin/streptomycin and incubated at 37°C for 25 min. ROS mediate the conversion of this compound to fluorescent DCF, which was then detected by an Epoch microplate reader (BioTek Instruments, Inc., Winooski, VT) at the compound’s excitation/emission spectra of approximately 492–495/517–527 nm.

### Hematopoietic Stem Cell Transplantation to Generate Chimeric Mice

To reduce the nonspecific effects of irradiation on the spinal cord, a preliminary dosing study was performed. Wild-type C57BL6 mice were exposed to a split dose of 8, 10.5, or 13 Gy of radiation from a Cesium-137 radioactive core. The two half doses were separated by 3 hours. Hematopoietic stem cells (HSCs) were isolated from Actin-GFP donors, and 1×10^6^ HSCs were administered by retro-orbital injection into recipients one hour after the last dose of radiation. Mice were maintained on water supplemented with antibiotics (40 mg sulfamethoxazole/8 mg trimethoprim per 100 ml water) (Bactrim) for one week prior to and 4 weeks after irradiation. Eight weeks after hematopoietic stem cell transplantation (HSCT), chimerization efficiency was determined by cytofluorometric analysis of circulating leukocytes. Whole blood was collected via cardiac puncture. Following the removal of erythrocytes by hyperosmotic lysis in ammonium chloride and Fc blocking (BD, 553142), leukocytes were stained with CD45-APC (BD Pharmingen, 559864), CD11b (Biolegend, 101235), and Ly6G (BD Biosciences, 560601). Stained blood samples were analyzed using a BD FACSymphony, and analysis was performed with FlowJo software. Additionally, the number of GFP + cells was assessed in coronal sections of the thoracic spinal cord. As described in the results, 10.5 Gys is the optimal dose that maximizes chimerization efficiency while minimizing leukocyte spinal cord infiltration. Therefore, a 10.5 Gy dose was utilized for subsequent HSCT studies. To generate cPLA2 KO chimeras, HSCT was performed as described above. cPLA2 KO and WT littermates were used as donors. Animals were injured 8–15 weeks after HSCT. Chimerization efficiency was determined at study endpoints as described below via qRT–PCR.

### Spinal Cord Injury

As described previously [31], mice were anesthetized via intraperitoneal injection of ketamine (100 mg/kg) and xylazine (10 mg/kg). Following a T9 laminectomy, mice received a 65 kDyn T9 contusion SCI (Infinite Horizons Impactor) [32]. Based upon a priori exclusion criteria, any mouse receiving SCI with abnormalities in the force vs. time curve generated by the IH device was considered to have received a “bad hit” and was excluded from analyses (see Table 1). Abnormalities meriting exclusion include bone hits or instability in the spinal cord at the time of injury. After injury the muscle incision was closed with an absorbable suture and the skin incision was closed using a monofilament suture. Mice received buprenorphine analgesic (Buprenex SR, 1.0 mg/kg) and Baytril antibiotic (Enrofloxacin, 5.0 mg/kg) subcutaneously once immediately after surgery as well as saline (1.0 mL) and antibiotic (5 mg/kg) subcutaneously for 5 days following surgery.

### Behavioral Assessment of Locomotor Recovery

Locomotor recovery was assessed after SCI with the Basso Mouse Scale (BMS) open field test, CatWalk XT gait analysis system, and the horizontal ladder test. The BMS utilizes a 9-point rating scale to characterize gross locomotor functions ranging from complete paralysis (score 0) to normal functions (score 9) as mice explore an open field for 4 minutes [33]. BMS scores were obtained at 1, 3, 7, 14, 21, 28, 35, and 42 dpi by two observers blinded to the treatment groups. Each hindlimb was scored separately based on movement (e.g., ankle placement and stepping), coordination, and trunk stability. Averaging both hindlimb scores generated a single score for each mouse. BMS subscores were derived from observations made during BMS scoring as described by Basso et al. [33]. BMS subscores are based on features of locomotion, such as trunk stability, coordination, and paw placement, that are observable in higher-functioning mice. BMS subscores permitted a better resolution to differentiate between mice with higher BMS scores.

We also used the CatWalk XT gait analysis system (Noldus, Wageningen, the Netherlands) to track specific parameters of gait. For CatWalk analysis, mice underwent three testing sessions: one week before injury and 4- and 6-weeks post-injury. The CatWalk features a red overhead light and green illuminated walkway, which reflects light in response to the contact of the mouse’s paw that is then captured via calibrated video recordings. Gait analysis was performed by the same researcher in a dark room. Using conditioning and analysis protocols developed previously [34], mice were allowed to acclimate in the room for 30 minutes prior to testing. For a single run, the mouse was first placed in the open end of the CatWalk under the red ceiling light and allowed to walk across the walkway. Each mouse completed three continuous runs on each analysis day, and a minimum of three valid runs, or complete walkway crossings, were obtained for each subject. Trials in which the mouse stopped, turned around, or significantly changed its speed during a run were excluded from analysis. Mice that did not complete three continuous runs after 25 attempts were excluded from analysis. Runs were analyzed by one researcher who was blinded to group assignments.

The horizontal ladder test was used to assess stepping and coordination at later stages of recovery according to previous studies [35, 36]. Mice were recorded from below with a high-resolution camera at 60 frames per second as they traversed 50 rungs of a metal ladder positioned horizontally. The ladder has 4 mm diameter rungs spaced 12 mm apart. A dark escape box was placed at the end of the 50th rung. An observer blinded to group inclusion watched the recordings and counted total hind paw slip events and total hind paw steps. Paw slips were counted when a hind paw fell below the rungs of the ladder at any point during a step cycle. Hind paw steps were counted any time a mouse restarted their step cycle. Paw slips were normalized to total steps taken by either hind paw and expressed as a percent. Three trials were quantified for each mouse, and the average of the 3 was used for the individual mouse. Trials were analyzed by one researcher who was blinded to group assignments.

### Immunohistochemistry

At the study endpoint, mice were given a lethal dose of ketamine and xylazine. Blood was then collected by cardiac puncture in a heparinized needle and syringe and transferred to an EDTA-coated tube (VWR, 101094–004) for leukocyte isolation (see below). Mice were transcardially perfused with PBS followed by 4% paraformaldehyde (PFA) in PBS (Millipore sigma). A 12 mm section of the spinal cord was collected that spanned the T9 lesion site. The spinal cord sections were postfixed in 4% PFA for 2 hours and washed overnight in 0.1 M PB. Spinal cords were dehydrated in 30% sucrose for 1 week. Six millimeters of spinal cord tissue centered on the lesion site was blocked in optimal cutting temperature compound (OCT) (Sakura FineTek, USA, 4583). Each block contained 5 spinal cords (2–3 randomly selected per group). Spinal cords were serially sectioned coronally at a thickness of 10 μm in the coronal orientation. Tissue was collected on ColorFrost Plus Microscope Slides (Fisher Scientific). Ten sets of tissue were generated for each block that spanned the length of the lesion, and the distance between each section on a single slide was 100 μm.

To assess lesion volume, all tissue was stained with eriochrome cyanine and neurofilament heavy, which stain intact myelin and axons, respectively. These markers were used to distinguish lesioned from intact tissue. To stain for neurofilament, sections underwent antigen retrieval in citrate buffer (pH 6.0) at 80°C for 5 minutes. Sections were then treated with 0.3% hydrogen peroxide in 40% methanol and PBS to quench endogenous peroxidase activity. Next, sections were blocked in 5% normal goat serum in PBS with 0.1% Triton-X 100 for 1 hour. Sections were stained overnight at 4°C with neurofilament-200 kD (1:1,500: Ck x NF200; NFH; Aves Labs). Sections were washed in PBS followed by a 1-hour incubation with biotinylated goat anti-chicken (1:2000, BA9010, Vector Laboratories). Sections were then incubated in avidin-biotin complex solution (ABC; 1:200; PK-6100; Vector Laboratories) and developed using 3,3’-diaminobenzidine (DAB). Sections were counterstained with eriochrome cyanine to visualize spared white matter. Stained slides were dehydrated using graded ethanol dilutions, cleared using Histoclear (101412–878; VWR Scientific), and coverslipped using Permount (SP15–500; Fisher Scientific). Slides were imaged using Axioscan (model Z1, Carl Zeiss AG., Oberkochen, GE) at 20x magnification and visualized and quantified using Halo software (Indica Labs, Albuquerque, NM).

### Gene Expression Analysis

At the study endpoint, peripheral blood was collected from all mice via cardiac puncture. Erythrocytes were removed using red blood cell (RBC) lysis buffer (BioLegend, 420301). Collected blood was mixed with RBC lysis buffer and left at room temperature for 5 minutes. Leukocytes were pelleted by centrifugation at 350 g for 5 minutes, and the lysis process was repeated twice. Leukocyte pellets were frozen on dry ice. RNA was isolated from leukocyte pellets using the RNeasy mini kit (Qiagen, 74104). RNA concentration was determined using a Nanodrop (Nanodrop Lite; Thermo Fisher Waltham, MA). cDNA libraries were made using a High-Capacity cDNA Reverse Transcription Kit (Applied Biosystems, 4368814). qRT–PCR was performed using SYBR green master mix and the following forward and reverse primers: (pla2g4a forward= “GATTCTGGAAATGTCTCTGGAAG”, reverse=”GGCTGACATTTTTCATTAGCTC”; GAPDH, forward=”CATCACTGCCACCCAGAAGACTG “, reverse=”ATGCCAGTGAGCTTCCCGTTCAG “).

NanoString gene expression analysis was performed on whole spinal cord homogenate. Seven days after SCI, mice were transcardially perfused with PBS, and 6 mm of the spinal cord was removed and flash-frozen in liquid nitrogen. RNA was isolated from spinal cords using an RNeasy mini kit (Qiagen, 74104). RNA concentration was determined using a NanoDrop Lite (Thermo Fisher Waltham, MA), and samples were diluted to 15 ng/μl. RNA analysis was performed using Nanostring nCounter (nCounter SPRINT profiler; NanoString Technologies; Seattle, WA), available at the University of Kentucky’s Genomics Core Laboratory. The neuroinflammation code set was used to interrogate expression changes in genes associated with secondary injury processes that occur after SCI.

### Statistics

All statistical tests were performed using GraphPad Prism software (v9.4.1, Boston, MA). All *in vitro* data were analyzed using one- or two-way ANOVA followed by Dunnett’s test for multiple comparisons. An unpaired Student’s t test was used to compare cPLA2 expression between cPLA2 KO and WT chimeras. For behavioral data and histological analysis, a two-way repeated-measures ANOVA was used. Behavioral data were compared over time, and histological data were compared over distance from the epicenter. *p* < 0.05 was considered significant.

## Results

### cPLA2 is required for myelin to potentiate an inflammatory stimulus in bone marrow-derived macrophages:

**Figure 1:**
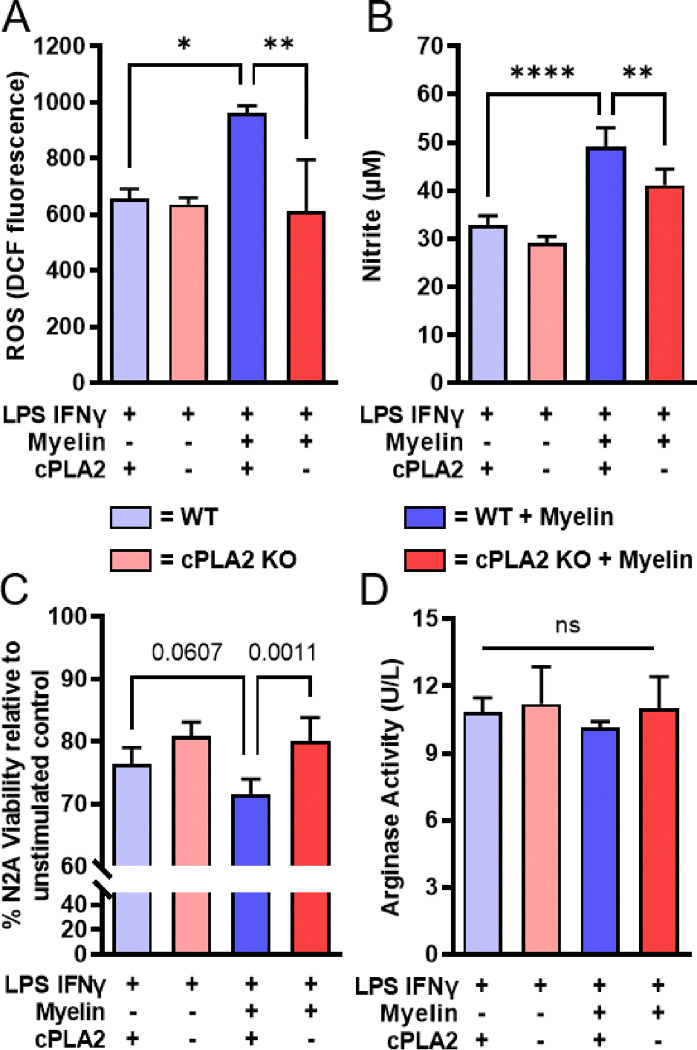
cPLA2 KO bone marrow-derived macrophages (BMDMs) have normal responses to proinflammatorystimuli but are resistant to the potentiating effect of myelin. Bone marrow harvested from cPLA2 KO mice and WT littermates was differentiated into BMDMs and treated with an M1 stimulus (LPS and IFN-γ) with or without myelin co-stimulation. **(A-B)** cPLA2 KO macrophages produce less ROS and nitric oxide following combined M1+myelin stimuli than WT macrophages. **(C)** An MTT assay measurement of N2a cell viability was used to determine the neurotoxic potential of macrophage media. Macrophage-conditioned media (MC) from cPLA2 KO macrophages stimulated with M1+myelin were less neurotoxic than MCM fromWT macrophages. **(D)** cPLA2 KO and WT macrophages had similar arginase activity across treatment groups. Data represent 3 biological replications of both BMDMs and myelin source. A n=3/group, B n=4/group, C n=5/group, D n=3/group. One-way ANOVA with Tukey’s multiple comparisons test. *p<0.05, **p<0.01, ***p<0.001, ****p<0.0001.

In the context of SCI, bone marrow-derived macrophages (BMDMs) are bombarded by an array of stimuli that potentiate a proinflammatory phenotype [3]. In addition, myelin debris is present at the injury site. Previously, we determined that BMDMs *in vitro* can be used to predict intraspinal macrophage responses [37]. Here, we employed an *in vitro* model of BMDMs derived from cPLA2 KO and WT mice to investigate the role of cPLA2 in combined proinflammatory (LPS + IFNγ or M1) and myelin stimulation. No significant differences were noted between cPLA2 and WT cells under control conditions (M1 stimuli alone, Fig. 1). As we reported previously [8], BMDMs produced significantly more reactive oxygen species (ROS) and nitric acid when stimulated with M1 stimuli in combination with myelin than when stimulated with M1 stimuli alone (Fig. 1A–B). This combinatorial effect of M1 + myelin on ROS and nitric acid production was significantly decreased in cPLA2-deficient macrophages compared to WT controls (Fig. 1A–B). Macrophage-mediated neurotoxicity was assessed by measuring the viability (MTT assay) of N2A neurons treated with macrophage-conditioned media (MCM) isolated from stimulated BMDMs. MCM from macrophages treated with M1 + myelin was more neurotoxic than M1-stimulated macrophages, however this trend did not reach significance (Fig. 1C). MCM from cPLA2-KO M1 + myelin-stimulated macrophages was significantly less neurotoxic than that from WT controls stimulated with M1 + myelin (Fig. 1C). Arginase activity was unchanged in all groups regardless of cPLA2 genotype and treatment (Fig. 1D). Collectively, these data implicate cPLA2 in the myelin-mediated potentiation of proinflammatory and neurotoxic macrophage activation.

### Optimization of Radiation Dose for the Generation of Chimeric Mice

**Figure 2:**
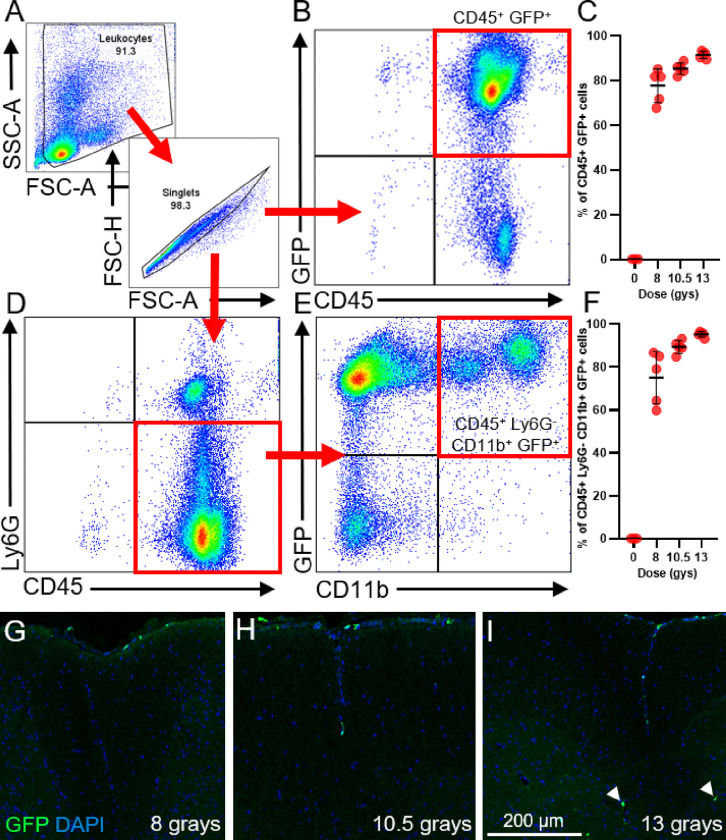
Hematopoietic stem cell transplantation (HSCT) at specific radiation doses produces robust leukocyte chimerism while minimizing leukocyte spinal cord infiltration. Uninjured mice received 8, 10.5, and 13 Gy of radiation in two doses following intravenous injection of HSCs from Actin-GFP donors. Chimerization efficiency and spinal cord leukocyte infiltration were determined 9 weeks post-HSCT. **(A)** Gating strategy used to determine the chimerization efficiency of circulating CD45^+^ leukocytes **(B)** and CD45^+^ CD11b^+^ Ly6G^−^ myeloid cells **(D-E)** at different radiation doses. **(C)** The percentage of CD45^+^ leukocytes that are GFP^+^ demonstrates a dose response to radiation and chimerization efficiency. **(F)** The percentage of CD45^+^ Ly6G^−^ CD11b^+^ cells that are GFP^+^ also demonstrates a dose-response effect of radiation dose on chimerization efficiency. **(G-I)** GFP^+^ cells in the spinal cord 9 weeks post-HSCT. A higher radiation dose increases intraparenchymal GFP^+^ cells in the spinal cord (arrowheads), while lower doses limit GFP^+^ cells to the meninges. Images representative of n=4–5/group. Scale bar is 500 μm. n=3 for control and n=5/group for 8, 10.5, and 13 gys.

To test whether cPLA2 potentiated proinflammatory macrophage activation *in vivo*, we utilized a chimeric mouse model. We first performed a pilot experiment to optimize the radiation dose in our HSCT protocol. Our goal was to achieve robust bone marrow chimerization while minimizing immune cell infiltration into the intact CNS. Hematopoietic stem cells (HSCs) isolated from Actin-GFP mice were retro-orbitally injected into C57BL/6J mice after three different radiation doses (8, 10.5, and 13 Gy). Ten weeks after HSCT, leukocyte chimerization and spinal cord immune cell infiltration were assessed using flow cytometry and immunofluorescence microscopy, respectively. The proportion of GFP^+^ CD45^+^ leukocytes and GFP^+^ CD45^+^ CD11b^+^ Ly6G^−^ myeloid cells increased in a dose-dependent manner (Fig. 2A–F). Additionally, GFP^+^ cells in the spinal cord parenchyma also increased in a dose-dependent manner with the highest radiation dose (13 Gy), producing noticeably more intraparenchymal GFP^+^ immune cells in the uninjured spinal cord compared to mice that received 10.5 Gys (Fig. 2G–I). In summary, the 10.5 Gy dose achieved over 90% chimerization of circulating leukocytes (Fig. 2C) and myeloid cells (Fig. 2F) with minimal spinal cord infiltration. Therefore, 10.5 Gy of radiation was used in subsequent chimera experiments.

### Locomotor Recovery and Tissue Pathology are Not Altered in cPLA2 KO Chimeras

**Figure 3:**
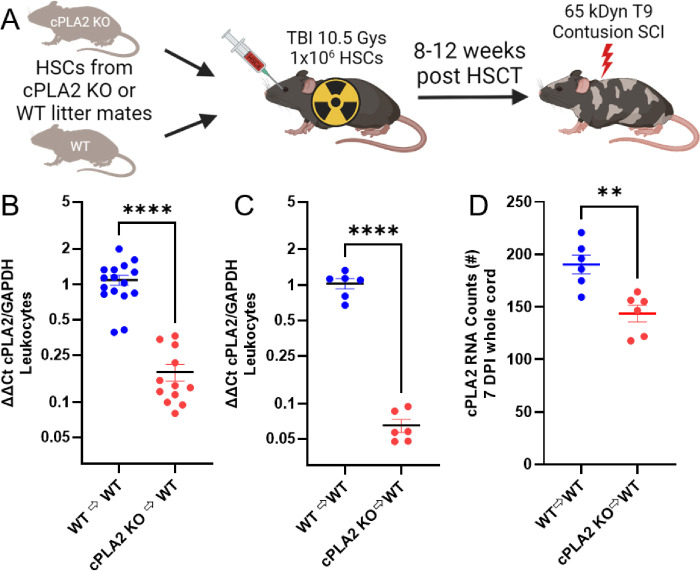
HSCT with cPLA2 KO donors reduces cPLA2 expression in circulating leukocytes and in the injured spinal cord. **(A)** Schematic for the HSCT protocol. HSCs isolated from cPLA2 KO and WT donors wereinjected into WT (C57BL/6J) mice after a split dose of 10.5 Gys. At 8–12 weeks after HSCT, chimerized mice are given a T9 65 kDyn contusion SCI. **(B, C)** (note the logarithmic scale) cPLA2 expression is decreased 10-fold in circulating leukocytes following HSCT. Each graph represents mice from one cohort. (**B**, WT n=16 KO n=12; **C**, WT n=6 KO=9) **(D)** cPLA2 RNA counts were decreased in whole spinal cord homogenate at 7 DPI. (n=6/group). (C-E=Welch’s t test), (F= unpaired t-test). *p<0.05, **p<0.01, ****p<0.0001.

Our optimized HSCT protocol enabled the interrogation of the role of cPLA2 in MDMs after SCI. WT C57BL6/J mice received HSCs from cPLA2 KO and WT littermate donors after 10.5 Gys. Eight to 12 weeks after HSCT, mice received a 65 kDyn T9 contusion SCI, and tissue was collected 7 days or 6 weeks after SCI (Fig. 3A–B). Whole blood was collected at the study endpoints, and cPLA2 expression was measured in isolated leukocytes. In all three cohorts, cPLA2 expression was decreased approximately tenfold in animals receiving KO vs. WT HSCs (Fig. 3B–C) (B, *p* < 0.0001; C, *p* < 0.0001). Additionally, the selective KO of cPLA2 in infiltrating MDM was sufficient to significantly reduce overall cPLA2 expression in the spinal cord at 7 days, further confirming the effectiveness of our KO approach (Fig. 3D) (*p* = 0.003).

**Figure 4:**
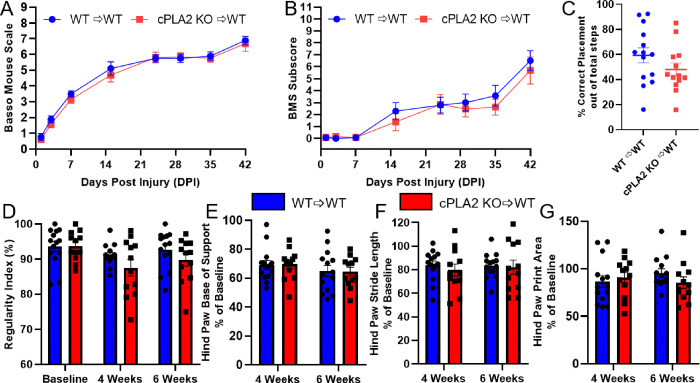
HSCT with cPLA2 KO donors did not alter locomotor recovery following SCI relative to WT controls. Mice were injured 10 weeks after HSCT, and recovery was assessed for 6 weeks after SCI. **(A-B)** Basso mouse scale locomotor scores and subscores were not significantly different between WT and cPLA2 KO HSC recipients. **(C)** Locomotor recovery and proprioception wereassessed at 6 weeks using the horizontal ladder test, and there was no significant difference between WT and cPLA2 KO recipients. **(D-G)** CatWalk gait analysis revealed no differences between groups. **(D)** Regularity index, **(E)** hind paw base of support width, **(F)** hind paw stride length, and **(G)** hind paw print area were assessed 1 week prior to injury and 4 and 6 weeks postinjury. **(E-G)**Data areexpressed as thepercent change from each mouse’s individual baseline parameter values. (A-C WT n=14 cPLA2 KO n=13) (D-G, WT n=14, cPLA2 KO n=12).

To test the hypothesis that MDM cPLA2 limits locomotor recovery after SCI, we assessed locomotor recovery of cPLA2 KO chimeras and WT chimera controls using the BMS score, BMS subscore, horizontal ladder, and CatWalkXT gait analysis system. BMS scoring and subscoring indicated recovery from complete paralysis with only some ankle movement (a score of 1 and 2) at 1 day postinjury (DPI) to frequent and consistent plantar stepping with coordinated stepping by 6 weeks postinjury (WPI) (a score of 6–8). BMS subscoring allowed discrimination between higher-functioning animals and demonstrated significant locomotor recovery over the six-week observation period. Both the BMS scores and subscores observed at 4 WPI were consistent with previous studies in unirradiated mice that received a moderate SCI [36]. Contrary to our hypothesis, neither BMS scoring nor subscoring indicated altered locomotor recovery in cPLA2 KO chimeras compared to WT chimera controls (Fig. 4A–B) (BMS scores, F_(1,25)_ = 0.268, *p* = 0.609; subscores, F_(1,25)_ = 0.3616, *p* = 0.553). Since these mice were capable of weight-supported stepping, we performed horizontal ladder beam testing to assess hind limb recovery, as it is more sensitive to the recovery of stepping and coordination than BMS scoring alone [35]. Horizontal ladder testing was performed only once at 6 WPI to limit adaptation and training to the test. Assessment of hindlimb function with horizontal ladder beam testing demonstrated no significant differences in locomotor recovery between cPLA2 KO and WT chimera controls (Fig. 4C) (*p* = 0.172).

CatWalk gait analysis was performed before injury and 4- and 6-weeks post-injury. Using the CatWalk XT, we specifically assessed the regularity index, base of support, stride length, and print area as continuous measures sensitive to moderate and severe SCI. The regularity index, a measure of coordination, was significantly decreased after 4 weeks (*p* = 0.003) but increased to almost preinjury levels after 6 weeks, mirroring the increase in BMS score and subscore in the same time frame. cPLA2 KO chimeras did not have a significantly different regularity index compared to the WT control at any of the three-time points assessed (Fig. 4D) (F_(1,24)_ = 1.806, *p* = 0.192). The hind paw base of support measures the distance between hind paws during one step cycle and was decreased at both 4 and 6 WPI. It was not significantly different between groups at either time point (Fig. 4E) (F_(1,24)_ = 0.1700, *p* = 0.684). Hind paw stride length measures the distance a single paw traverses between steps. Although hind paw stride length was relatively stable between 4 and 6 WPI, there was no significant difference between cPLA2 KO chimeras and WT controls (Fig. 4F) (F_(1,26)_ = 0.943, *p* = 0.340). Last, the hind paw print area measures the maximum paw print area during a placement event. The hind paw print area did not decrease after injury and was not different between groups at either timepoint (Fig. 4G) (F_(1,24)_ = 0.2100, *p* = 0.651).

**Figure 5:**
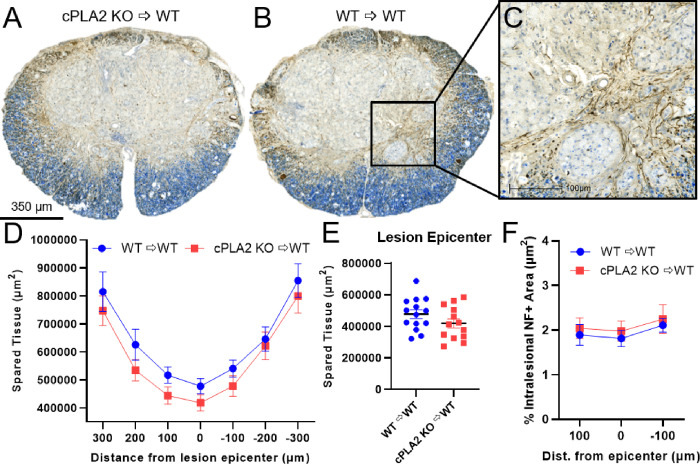
cPLA2 ablation in infiltrating leukocytes does not alter tissue sparing or intralesional axons after SCI. **(A, B)** Representative sections of the lesion epicenter stained for neurofilament (axons-brown) with an eriochrome cyanine (blue-myelin) counterstain 6 weeks after SCI. (**C**) High-powered image of B indicative of typical axonal labeling (brown) within the lesion epicenter and quantified in (F). cPLA2 KO and WT recipients had similar tissue sparing throughout the lesion **(D)** and at the lesion epicenter **(E)**. **(F)** A quantitative assessment of axon sparing/sprouting at the lesion epicenter and 100 μm caudal and rostral to the epicenter revealed no significant difference between WT and cPLA2 KO BM recipients. WT n=14, cPLA2 KO n=13.

At the end of the 6 weeks, tissue was collected to assess the effect of cPLA2 KO chimerism on tissue pathology after SCI. Using eriochrome cyanine (EC) and neurofilament heavy (NF) staining, spared tissue was identified as double-positive (EC+, NF+) myelinated axons. Representative lesion epicenter images illustrate a clear contrast between areas of spared tissue and frank lesion areas in spinal cord tissue (Fig. 5A–B). Tissue sparing was not significantly different at the lesion epicenter or rostral and caudal to the epicenter between cPLA2 KO and WT chimeras (Fig. 5D–E) (5E, *p* = 0.148; 5D, F_(1,25)_ = 1.382, *p* = 0.251). We also quantified intralesional axonal sprouting because other studies that manipulate MDMs in SCI have shown that intralesional axons are affected [38]. We measured intralesional axon sprouting by quantifying the percent of total neurofilament-positive area in the lesion epicenter and at 100 μm rostral and caudal to the epicenter (Fig. 5C). There was no difference in intralesionally spared axons between cPLA2 KO and WT chimeras in the sections assessed (F_(1,26)_ = 0.332, *p* = 0.569) (Fig. 5F).

## Discussion

The inflammatory microenvironment that persists chronically in the injured spinal cord limits recovery. The underlying mechanisms driving inflammation after SCI are poorly understood. Lipid-debris-laden macrophages that chronically linger in the injury site may be the culprit [5, 39]. Previous studies by our lab and others have shown that myelin can induce a proinflammatory macrophage phenotype [8, 40]. Our previous work suggests that PLA2 enzymes are crucial for myelin-induced potentiation of a proinflammatory macrophage phenotype. In this study, we confirmed our previous *in vitro* work using cPLA2 KO macrophages in lieu of a nonspecific chemical inhibitor (PACOCF_3_) [8] thus confirming that cPLA2 alone was sufficient to potentiate myelin-mediated proinflammatory activation. *In vivo*, cPLA2 KO chimeric mice had similar recovery and tissue sparing after SCI compared to WT chimera controls. Our findings suggest that cPLA2 does not have the same role in MDMs *in vivo* as it does in our *in vitro* model of the lipid-rich environment of SCI.

cPLA2 is widely studied in multiple diseases and is a key enzyme in arachidonic acid production [41]. As such, cPLA2 has been a potential therapeutic target for SCI for almost two decades. Several studies have used pharmacologic and genetic techniques to inhibit cPLA2 with varied results. The earliest report by Huang et al. administered a single dose of cPLA2 inhibitor, AACOCF_3_ after SCI and demonstrated improved locomotor recovery and neuronal survival during the first week [25].

Next, a comprehensive study of all PLA2s upregulated after SCI by Lòpez-Vales et al. reported reduced behavioral recovery and exacerbated tissue pathology after cPLA2 inhibition using a selective and potent 2-oxoamide-based inhibitor and a cPLA2 global knockout mouse [26]. This report also suggested that other PLA2s, such as secretory and calcium-independent phospholipases (sPLA2 and iPLA2), were pathological and that cPLA2 activity was beneficial. Work from the laboratory of Dr. Xiao-Ming Xu next reported that cPLA2 inhibition by continuous AACOCF_3_ administration in rats and global genetic ablation in mice improved behavioral recovery and tissue sparing [17]. In later studies, his group demonstrated that cPLA2 inhibition reduced RhoA/Rho kinase activity and vice versa [42]. RhoA/Rho kinase is a major barrier to regeneration after SCI, as it leads to growth cone collapse and neurite retraction [43]. Recently, his lab reported that cPLA2 inhibition decreases motor neuron loss and muscle atrophy after SCI [44]. Last, Li et al. reported that AACOCF_3_ rescued dysfunctional autophagy after SCI [19]. Overall, these reports, along with our data, suggest a complex role of cPLA2 in secondary injury and neuropathology after SCI.

In SCI, MDM cPLA2 may not contribute to secondary injury because most of the active cPLA2 is in neurons, astrocytes, and microglia. Early studies identified cPLA2-positive neurons in specific brain regions and spinal cord motor neurons in uninjured rodent CNS with only faint signals in glia [23, 45]. Later studies reported the presence of cPLA2 immunoreactivity in rodent models of focal and global cerebral ischemia and amyotrophic lateral sclerosis, as well as brain tissue from Alzheimer’s disease [21, 46, 47]. cPLA2 was present in regions of neuronal death but also in surrounding reactive astrocytes and microglia [48]. In SCI, cPLA2 was thought to be expressed only in neurons, oligodendrocytes, and a subpopulation of microglia [24, 26]. Recently, more sensitive approaches to detect cPLA2 expression using RNA counting in sorted cells and single-cell transcriptomics have revealed cPLA2 expression in microglia, MDMs, and endothelial cells within the first week after injury [28, 49]. However, it is important to note that cPLA2 is activated by intracellular calcium and mitogen-activated protein kinase-mediated phosphorylation; thus, antibody and nucleic acid-based detection are not sufficient to determine cPLA2 activity and concomitant arachidonic acid production [41]. Thus, a cell-specific cPLA2 activity assay would determine the most likely target of cPLA2 inhibition after SCI. Alternatively, a “reverse chimera” could be generated in which WT bone marrow is transplanted into cPLA2 KO mice. These approaches would further clarify the role of cPLA2 in secondary injury after SCI.

Alternatively, MDM cPLA2 may be detrimental to recovery, but the beneficial effect of MDM cPLA2 deletion could be overshadowed by deletion of cPLA2 in other HSC-derived cells, namely, platelets. cPLA2 inhibition in platelets with AACOCF_3_ reduces the production of thromboxane A2, also called platelet-activating factor (PAF) [50, 51]. Additionally, cPLA2 null mice have decreased platelet aggregation and increased bleeding times [52]. Our cPLA2 KO chimeras most likely had cPLA2-deficient platelets [53]. cPLA2 KO platelets may have had limited efficacy in hemostasis acutely after SCI, leading to exacerbated hemorrhage. The extent of hemorrhage after SCI impacts recovery not only in rodents but also in humans. Hemorrhage measured by radiological imaging can predict outcomes in cases of SCI [54–57]. Therefore, any beneficial effects of MDM cPLA2 KO may have been countered by exacerbated hemorrhage. Future experiments may be able to limit nonspecific cPLA2 inhibition through the use of Cre-mediated selective deletion from microglia and MDMs.

Our results demonstrate that cPLA2 activation in MDMs likely does not contribute significantly to macrophage-mediated pathology after SCI. We first showed that cPLA2 KO macrophages are protected from the proinflammatory potentiating effect of myelin. Using bone marrow chimeras, we then investigated the role of cPLA2 in MDMs after SCI. We found no effect of cPLA2 KO HSCT on locomotor recovery or tissue pathology. This discovery sheds light on a series of heterogeneous observations using global KO or suppression of cPLA2 in SCI.

## Figures and Tables

**Table 1:** Experimental design of the three cohorts including: group size pre and post exclusion, sex, outcomes, endpoint, and exclusion criteria for all groups.

Cohort	n per group	Sex	Outcomes	Timepoint	Exclusion
A	Actin-GFP->WT 8, 10.5, 13 grays n = 5/group Control n = 3	F	Bone marrow chimerization efficiency and CNS leukocyte permeability	8 weeks post HSCT	None
B	All animals T9 SCI WT->WT = 16 cPLA2KO->WT = 16 post-exclusion WT->WT = 14 cPLA2KO- >WT = 13	F	Behavioral analysis of locomotor recovery: BMS, Horizontal Ladder, Catwalk gait analysis Tissue pathology: Spared tissue and intralesional axons	6 weeks	2 mice from WT group and 1 mouse excluded from cPLA2 KO group for meeting a *priori* exclusion criteria (BMS score ≥3 at 1 dpi) 2 mice from cPLA2 KO group excluded based on elevated leukocyte cPLA2 expression at endpoint.
C	All animals T9 SCI WT->WT = 6 cPLA2KO->WT = 6	F	Gene expression of spinal cord homogenate by nCounter (NanoString Technologies) gene array	7 days	None

## Data Availability

All raw data presented in figures and tables herein is available to the public through the Open Data Commons for Spinal Cord Injury (ODC-SCI). The ODC-SCI is a dedicated data sharing portal and repository for the field of SCI that enables the sharing of data with the public using a DOI. The ODC-SCI complies with the FAIR data principles to ensure that SCI data is Findable, Accessible, Interoperable and Reusable.

## Abbreviations

AACOCF3: Arachidonyl trifluoromethyl ketone
ANOVA: Analysis of Variance
CM-H2DCFDA: chloromethyl 2′,7′-dichlorodihydrofluorescein diacetate
cPLA2: cytosolic phospholipase A2
BMS: Basso Mouse Scale
CNS: Central Nervous System
DAB: 3,3’-diaminobenzidine
DPI: days post injury
Gys: Grays
EDTA: Ethylenediaminetetraacetic acid
HSC: hematopoietic stem cell
HSTC: hematopoietic stem cell transplant
MDM: Monocyte-derived macrophage
MTT: thiazolyl blue tetrazolium bromide
PACOCF3: Palmitoyl trifluoromethyl ketone
PB: Phosphate Buffer
PBS: Phosphate Buffered Saline
SCI: Spinal Cord Injury
WPI: weeks post injury
WT: Wild Type
